# Supplementary material for: Multi‐dataset identification of innovative feature genes and molecular mechanisms in keratoconus
Source: J Cell Mol Med. 2024 Sep 19;28(18):e70079. doi: 10.1111/jcmm.70079 (PMC11412914; doi:10.1111/jcmm.70079)
Supplement: Supplementary file 2 — Material S2. [file JCMM-28-e70079-s002.zip › 7.GSEA analysis/4.MYRF/KEGG_PEROXISOME.html]

Details for gene set KEGG\_PEROXISOME[GSEA]

|  || Dataset | MYRF.group.cls#h\_versus\_l |
| Phenotype | group.cls#h\_versus\_l |
| Upregulated in class | h |
| GeneSet | KEGG\_PEROXISOME |
| Enrichment Score (ES) | 0.18226641 |
| Normalized Enrichment Score (NES) | 0.7006781 |
| Nominal p-value | 0.851927 |
| FDR q-value | 1.0 |
| FWER p-Value | 1.0 |
Table: GSEA Results Summary

  

Fig 1: Enrichment plot: KEGG\_PEROXISOME      
 Profile of the Running ES Score & Positions of GeneSet Members on the Rank Ordered List

  

| SYMBOL | TITLE | RANK IN GENE LIST | RANK METRIC SCORE | RUNNING ES | CORE ENRICHMENT || 1 | FAR2 | na | 638 | 0.533 | 0.0405 | Yes |
| 2 | DDO | na | 710 | 0.515 | 0.0953 | Yes |
| 3 | PEX11G | na | 1911 | 0.364 | 0.1009 | Yes |
| 4 | ACSL6 | na | 2596 | 0.318 | 0.1163 | Yes |
| 5 | BAAT | na | 3032 | 0.293 | 0.1361 | Yes |
| 6 | HMGCL | na | 4769 | 0.229 | 0.1113 | Yes |
| 7 | ABCD4 | na | 5640 | 0.204 | 0.1087 | Yes |
| 8 | NUDT19 | na | 6022 | 0.192 | 0.1188 | Yes |
| 9 | PEX11A | na | 6080 | 0.190 | 0.1382 | Yes |
| 10 | SLC27A2 | na | 6392 | 0.181 | 0.1492 | Yes |
| 11 | MVK | na | 7199 | 0.158 | 0.1434 | Yes |
| 12 | ACOX1 | na | 7873 | 0.141 | 0.1396 | Yes |
| 13 | CRAT | na | 8487 | 0.127 | 0.1359 | Yes |
| 14 | PEX26 | na | 8826 | 0.120 | 0.1394 | Yes |
| 15 | PXMP4 | na | 8971 | 0.117 | 0.1481 | Yes |
| 16 | HAO1 | na | 9183 | 0.112 | 0.1544 | Yes |
| 17 | PEX12 | na | 9311 | 0.109 | 0.1628 | Yes |
| 18 | ECH1 | na | 9474 | 0.106 | 0.1699 | Yes |
| 19 | PEX2 | na | 10235 | 0.092 | 0.1581 | Yes |
| 20 | ACAA1 | na | 10411 | 0.088 | 0.1628 | Yes |
| 21 | ACOX3 | na | 10534 | 0.086 | 0.1687 | Yes |
| 22 | FAR1 | na | 10605 | 0.085 | 0.1761 | Yes |
| 23 | PECR | na | 10709 | 0.083 | 0.1823 | Yes |
| 24 | NUDT12 | na | 11162 | 0.075 | 0.1776 | No |
| 25 | PEX16 | na | 11834 | 0.066 | 0.1655 | No |
| 26 | ACSL1 | na | 11845 | 0.066 | 0.1724 | No |
| 27 | PMVK | na | 12132 | 0.062 | 0.1710 | No |
| 28 | CAT | na | 12267 | 0.060 | 0.1737 | No |
| 29 | SCP2 | na | 12531 | 0.056 | 0.1723 | No |
| 30 | XDH | na | 12948 | 0.051 | 0.1659 | No |
| 31 | PHYH | na | 13544 | 0.043 | 0.1535 | No |
| 32 | PEX1 | na | 13563 | 0.043 | 0.1577 | No |
| 33 | PEX11B | na | 13696 | 0.041 | 0.1585 | No |
| 34 | PEX10 | na | 13777 | 0.040 | 0.1607 | No |
| 35 | AGPS | na | 14243 | 0.035 | 0.1511 | No |
| 36 | GNPAT | na | 14722 | 0.030 | 0.1407 | No |
| 37 | PEX7 | na | 14790 | 0.030 | 0.1420 | No |
| 38 | HACL1 | na | 15268 | 0.025 | 0.1311 | No |
| 39 | MPV17 | na | 15380 | 0.024 | 0.1305 | No |
| 40 | EPHX2 | na | 15591 | 0.022 | 0.1269 | No |
| 41 | PRDX1 | na | 16051 | 0.017 | 0.1155 | No |
| 42 | PEX5 | na | 16478 | 0.013 | 0.1047 | No |
| 43 | PRDX5 | na | 17370 | 0.005 | 0.0796 | No |
| 44 | MLYCD | na | 17418 | 0.005 | 0.0787 | No |
| 45 | HSD17B4 | na | 18023 | -0.001 | 0.0614 | No |
| 46 | PAOX | na | 18790 | -0.008 | 0.0402 | No |
| 47 | PEX19 | na | 19086 | -0.011 | 0.0329 | No |
| 48 | ACOT8 | na | 19133 | -0.012 | 0.0329 | No |
| 49 | ABCD1 | na | 19623 | -0.017 | 0.0207 | No |
| 50 | GSTK1 | na | 19746 | -0.019 | 0.0193 | No |
| 51 | ACSL4 | na | 19912 | -0.021 | 0.0168 | No |
| 52 | ECI2 | na | 20501 | -0.028 | 0.0029 | No |
| 53 | PEX14 | na | 20561 | -0.028 | 0.0043 | No |
| 54 | AMACR | na | 21406 | -0.038 | -0.0158 | No |
| 55 | SOD1 | na | 21437 | -0.039 | -0.0124 | No |
| 56 | DHRS4 | na | 21442 | -0.039 | -0.0083 | No |
| 57 | IDH2 | na | 21549 | -0.040 | -0.0069 | No |
| 58 | PIPOX | na | 22212 | -0.048 | -0.0206 | No |
| 59 | SLC25A17 | na | 23166 | -0.062 | -0.0413 | No |
| 60 | EHHADH | na | 23596 | -0.069 | -0.0460 | No |
| 61 | ABCD3 | na | 23671 | -0.070 | -0.0404 | No |
| 62 | PEX3 | na | 23687 | -0.071 | -0.0330 | No |
| 63 | IDH1 | na | 23974 | -0.075 | -0.0330 | No |
| 64 | PEX13 | na | 24566 | -0.085 | -0.0406 | No |
| 65 | SOD2 | na | 25176 | -0.096 | -0.0475 | No |
| 66 | ACSL5 | na | 25278 | -0.098 | -0.0396 | No |
| 67 | ACSL3 | na | 25434 | -0.102 | -0.0328 | No |
| 68 | DECR2 | na | 25475 | -0.102 | -0.0227 | No |
| 69 | PEX6 | na | 27256 | -0.141 | -0.0585 | No |
| 70 | CROT | na | 27384 | -0.144 | -0.0462 | No |
| 71 | AGXT | na | 28253 | -0.168 | -0.0527 | No |
| 72 | MPV17L | na | 28442 | -0.174 | -0.0389 | No |
| 73 | PXMP2 | na | 30078 | -0.226 | -0.0611 | No |
| 74 | ABCD2 | na | 31378 | -0.284 | -0.0672 | No |
| 75 | HAO2 | na | 31461 | -0.289 | -0.0377 | No |
| 76 | ACOX2 | na | 31851 | -0.309 | -0.0147 | No |
| 77 | DAO | na | 32460 | -0.347 | 0.0061 | No |
| 78 | NOS2 | na | 34080 | -0.537 | 0.0187 | No |
Table: GSEA details [plain text format]

  

Fig 2: KEGG\_PEROXISOME      
 Blue-Pink O' Gram in the Space of the Analyzed GeneSet

  

Fig 3: KEGG\_PEROXISOME: Random ES distribution      
 Gene set null distribution of ES for **KEGG\_PEROXISOME**
